# The Relationships of Self-Esteem, Future Time Perspective, Positive Affect, Social Support, and Career Decision: A Longitudinal Multilevel Study

**DOI:** 10.3389/fpsyg.2018.00514

**Published:** 2018-04-26

**Authors:** In-Jo Park, Minhee Kim, Seungwoo Kwon, Hae-Gyoung Lee

**Affiliations:** ^1^Department of Psychology, Henan University, Kaifeng, China; ^2^Korea Counseling Graduate University, Seoul, South Korea; ^3^Business School, Korea University, Seoul, South Korea; ^4^Department of Youth Coaching & Counseling, Korea Soongsil Cyber University, Seoul, South Korea

**Keywords:** career decision-making self-efficacy, career choice anxiety, self-esteem, self-efficacy, social support, emotional regulation

## Abstract

This study aimed, first, to determine whether the intra-individual variability in positive affect was related to the intra-individual variability in career decision-making self-efficacy, and career choice anxiety. The second objective was to examine whether social support moderates the relationship between affect and these outcome variables. The third objective was to find out how career decision-making self-efficacy and career choice anxiety change according to self-esteem and future time perspective. We conducted a study using the daily diary method in which participants were asked to rate their affect or attitudes for 21 consecutive days. In total, 128 university students participated in this study. The main results were as follows. First, positive affect was associated positively with career decision-making self-efficacy and negatively with career choice anxiety. Second, social support had a synergy effect with positive affect to influence career choice anxiety. Third, self-esteem was related positively to career decision-making self-efficacy and negatively to career choice anxiety. We discuss theoretical and practical implications.

## Introduction

Most young adults are well aware of the significance of career decision-making and are very concerned about their future careers. While attempting to make a good career decision, each individual must consider his or her own preferences, interests, capabilities, and skills (Judge et al., 2010; Ng and Feldman, 2010). In addition, external factors—career prospects, variations in the job market, etc.—can influence career decision-making. In Korea, since 2015, the unemployment rate for 20–29-year-olds was 9–10% because of the economic downturn (Korean Statistical Information Service, 2017). Therefore, college graduates have difficulty in getting the jobs they want (Joo, 2014). All these hardships can result in confusion and difficulties in career decision-making (Levin and Gati, 2015). Thus, it is necessary to conduct studies to understand the process of young peoples’ career decision-making and to assist those who are experiencing these difficulties.

Social cognitive career theory (SCCT; Lent et al., 1994; Lent and Brown, 2013) is the most influential career theory that could explain the process of career decision-making and career-related attitude development. According to SCCT, self-efficacy expectancy and outcome expectations are crucial factors that directly or indirectly affect career-related outcome expectations, goals, behaviors, and performance (Thompson et al., 2017). Based on SCCT, this study focused on self-efficacy related to career decision-making, namely career decision-making self-efficacy. In addition, anxiety is a typical indicator of negative outcome expectation experienced when people perform in a given domain; therefore, we also focused on anxiety related to career decision-making—namely, career choice anxiety. Career choice anxiety can be viewed as a consequence of outcome expectations, as it is in some studies (e.g., Brown et al., 2014). However, the theory of the relationship between emotion and cognition (Phelps, 2006; Pessoa, 2008) indicates that, while emotion such as anxiety is the consequence of cognition such as outcome expectations, emotions also affect expectations. Because it is difficult to separate emotion from cognition, in the current study, we considered as an outcome expectation career choice anxiety, which is an emotional indicator of negative outcome expectation for career decision-making.

Examining career decision-making self-efficacy and career choice anxiety can provide more insight into the career decision-making process. People with high self-efficacy for career decision-making can develop career plans effectively, but those with a high level of career choice anxiety may experience difficulties in the career decision-making process (Germeijs et al., 2006). In other words, those with high career decision-making efficacy can make successful career decisions and perform successfully in their careers whereas those with high career choice anxiety may experience negative outcomes in career decision-making and career performance as they are overwhelmed by the career decision-making process. A study on career discontinuity and reemployment found that the most prominent difficulty faced by women with career disconnection was the loss of self-efficacy regarding making new career decisions (Gwal, 2016). Based on this finding, it is necessary to identify individual factors that affect the career decision-making process, such as career choice anxiety (e.g., Rottinghaus et al., 2009).

Among the processes involved in the SCCT model, the present study focused on the relationship between self-efficacy expectancy and outcome expectation with proximal variables (individual and contextual influences). More specifically, we investigated career decision-making self-efficacy as self-efficacy expectancy, career decision choice anxiety as outcome expectation, self-esteem and future time perspective as inter-individual variability, positive affect experience as intra-individual variability, and social support as a contextual influence.

Previous studies (e.g., Bruine de Bruin et al., 2007; Jin et al., 2009) have focused on inter-individual differences affecting the career decision and career development processes. However, some career development researchers (e.g., Feldman, 2002; Abele and Spurk, 2011) were more interested in what happens within an internal state of individual—his or her affect—that influences the career decision-making process. An individual’s affective state can be the key to understanding the appropriate career intervention plan in the career-counseling domain (Hirschi and Freund, 2014). In particular, Germeijs et al. (2006) argued that a diary-based approach is needed rather than a questionnaire response in order to fully understand the career decision process and to address the limitations of their longitudinal study. However, to our knowledge, no study has been conducted to investigate the fluctuations in career decision attitudes such as career decision-making self-efficacy and career choice anxiety on a daily basis. Recently, career adaptability or job performance research using a daily diary method has shown that job and career satisfaction (Zacher, 2015), transformational leadership, job engagement, self-efficacy, and optimism (Tims et al., 2011) can change daily. Jung et al. (2015), using ESM (Experienced Sampling Method), showed that career decision-making self-efficacy and career choice anxiety can change over 7 days.

It is also well known that anxiety can vary by state (Spielberger, 1971; Marteau and Bekker, 1992). Unlike trait anxiety, state anxiety may fluctuate depending on the situation and is a function of the stressors (Barnes et al., 2002). Weinstein et al. (2002) indicated that career choice anxiety may depend on the degree of perceived control over the situation. In line with previous studies (Weinstein et al., 2002; Tims et al., 2011; Jung et al., 2015; Zacher, 2015), career decision-making self-efficacy and career choice anxiety may be viewed as a state that may fluctuate within the person. Based on this, we assumed that career decision-making self-efficacy and career choice anxiety would change daily.

Therefore, the present study focused on the roles of proximal variables (inter/intra-individual differences and a contextual influence variable) in career decision-making self-efficacy and career choice anxiety, both of which are important variables in the career decision-making process according to SCCT. Thus, the first purpose of this study was to determine whether the intra-individual variability in positive affect is related to the intra-individual variability in career decision-making self-efficacy and career choice anxiety. We conducted a study using the daily diary method in which participants were asked to rate their affect or attitudes for 21 consecutive days. The second objective was to examine whether social support regulates affect and these outcome variables. The third objective was to find out how career decision-making self-efficacy and career choice anxiety change according to self-concept—general self-efficacy and self-esteem—widely known as relatively stable individual differences. In other words, we investigated whether attitude toward career decision differs within 21 days according to the individual differences in general self-efficacy and self-esteem.

## Theoretical Background

### SCCT and Career Decision-Making Process

SCCT (Lent et al., 1994, 2000) is an integrative model that incorporates Bandura’s (1986) general social cognitive framework with concepts from earlier career development theories. SCCT supposes that links among social cognitive variables, person input, goal-related behaviors, and contextual factors contribute to career-related outcomes (Lent et al., 1994). Since SCCT was introduced, it has become a popular base for career research and intervention in the past two decades (Sheu and Bordon, 2017). It emphasizes the role of self-efficacy expectations and outcome expectations in the formation of career choice goals and career interests as well as career-related performance outcomes (Dickinson et al., 2017).

Self-efficacy refers to beliefs that people have about their abilities to plan and execute performance successfully (Bandura, 1997), which subsequently lead to the formation of career-related interests, choice goals, and performance. Since self-efficacy is domain-specific, people may have high self-efficacy in some domains (e.g., math) but low self-efficacy in others (e.g., sports). Self-efficacy stems from the learning experiences that include personal performance experiences, vicarious learning, persuasion from significant others, and interpretations of one’s own affective and physiological states when performing the tasks in a domain (Bandura, 2001).

Based on SCCT, career or vocational psychologists have been encouraged to explore how contextual and individual variables, such as career decision-making self-efficacy, affect the career development process of youth (Hackett and Byars, 1996). Many studies generally supported the application of the SCCT framework to career-decision research in that self-efficacy has influenced career choice, career indecisiveness, and career choice satisfaction, among others (Lent et al., 2003; Schaub and Tokar, 2005; Sheu et al., 2010). Further, SCCT has been found to be useful for accounting for the career development experiences in diverse populations (e.g., Byars-Winston et al., 2010; Lent et al., 2010, 2011). However, very few studies have tested the utility of the SCCT among Korean college students (Joo et al., 2015).

### The Influence of Positive Affect on Career Decision-Making Self-Efficacy and Career Choice Anxiety

Taylor and Betz (1983) defined career decision-making self-efficacy as a confidence or belief that one can successfully complete challenges necessary for career decision-making. Since then, researchers of the career decision-making process (e.g., Flores et al., 2006; Gushue et al., 2006) have been interested in career decision-making self-efficacy. In particular, career decision-making intervention studies have focused on improving career decision-making self-efficacy (e.g., Reese and Miller, 2006; Scott and Ciani, 2008). Studies have shown that improving career decision-making self-efficacy assists career decision-making by enhancing individuals’ career planning (Chung, 2002) and exploration activities (Gushue et al., 2006) and by reducing career indecision (Osipow, 1999).

Meanwhile, in the career decision-making process, the most prominent hindrance for career development is career choice anxiety. Career choice anxiety has been defined as emotional distress associated with career decision-making (Germeijs et al., 2006). It refers to difficulty in dealing with career-related information and making actions (Corkin et al., 2008). Individuals who have difficulty in career decision-making may experience career choice anxiety (Miller and Rottinghaus, 2014) and low performance in career decision-making tasks (Germeijs et al., 2006). Career decision-making self-efficacy and career choice anxiety are not polarized on one dimension; instead, they are two independent concepts. Therefore, studies on the career decision-making process need to examine these two variables separately.

Inspired by SCCT, many empirical studies have investigated the influence of individual difference variables (e.g., personality traits, affectivity, human resources, etc.) on career self-efficacy. Lent et al. (1994) SCCT suggested that individual difference variables play an important role in the development of career self-efficacy. One important intra-individual difference variable that may influence career decision-making self-efficacy and career choice anxiety is positive affect. According to the Broaden and Build Theory (Fredrickson and Joiner, 2002), experiencing positive affect (e.g., being happy, proud, and enthusiastic) extensively influences physical outcomes (e.g., immune system and longevity) and expands cognitive and behavioral repertoires (Fredrickson and Losada, 2005). Positive rather than negative affect is a strong predictor of health or functional abilities (Serafini et al., 2016).

Recently, positive affect has also been recognized as an individual variable that influences career-related outcomes (Haase et al., 2012). Although, so far, only a small number of studies have examined the role of positive affect, these studies have reported that positive affect is beneficial for making judgments about one’s own capacities across various domains of life (Sheldon et al., 1996). In Haase et al.’s (2012) longitudinal study, participants believed they had more control over achieving their goals when they experienced positive affect. In addition, Young et al. (2002) emphasized the role of affect on career development behaviors and found that affect is closely related to career development-related variables including individual goals, expectations, desires, and plans. They explained that affect influences career construction by motivating and restraining career-related behaviors and by promoting the development of career-related stories.

More specifically, positive affect can influence career decision-making self-efficacy. Positive affect can enhance self-efficacy since it is connected to success and the thought of overall well-being (Bandura, 1997). According to a previous study (Hammond et al., 2010), positive affect is an essential factor in career decision-making, and positive affect has a positive relationship with various self-efficacies related to career. Larson and Borgen (2006) found that positive affect was related to job confidence. Further, in their assessment of affect based on the characteristics affecting job exploration, Côté et al. (2006) found that positive affect had a positive relationship with job exploration clarity and were subsequently related to job exploration self-efficacy. Overall, these results indicate that positive affect influences career-related self-efficacy. In previous studies, positive affect was not measured on a daily basis. However, emotions are not stable and may vary often (Kidd, 2004; Hartung, 2011). Therefore, it is necessary to investigate the influence of positive affect experienced in daily life on the daily degree of career decision-making self-efficacy. The present study predicted that career decision-making self-efficacy would increase daily with an increase in the experience of positive affect.

Meanwhile, positive affect can lower career choice anxiety by arousing memories or beliefs related to success (Bandura, 1997). In the study by Albion and Fogarty (2002), while emotionally stable people were found to experience less difficulty in the career decision-making process, those who had low positive affect and lacked the skills to utilize affect tended to avoid career decision-making due to the decline in self-efficacy and, consequently, tended to experience greater difficulties in the career decision-making process. In other words, individuals were likely to be diffident and to experience anxiety regarding the career they had to choose when positive affect decreased. Considering all these factors together, the present study expected career choice anxiety to decrease daily with an increase in the experience of positive affect. Thus, the following hypotheses were established:

Hypothesis 1: Positive affect will be positively associated with career decision-making self-efficacy.
Hypothesis 2: Positive affect will be negatively associated with career choice anxiety.

### Relationships Among Positive Affect, Social Support, Career Decision-Making Self-Efficacy, and Career Choice Anxiety

Based on SCCT and the developmental and relational perspective, the contextual aspect—social context—must be considered to understand the optimal individual functioning within the context of career development (Lent and Brown, 2013). Even though many theories on career decision-making and career development traditionally focus on individuals, the awareness of the importance of social and relational contexts has been increasing over time (Hirschi and Freund, 2014). For instance, Blustein (2011) emphasized the relational context of career and asserted that the career decision-making exploration and its process can be promoted or restrained according to relationship experiences. In other words, social support can promote the career decision-making process by providing informational and emotional support toward career management through its application as a relational resource. Therefore, this study focused on the role of the relational context, particularly social support, to examine if it moderated the relationship between positive affect and career decision-making processes such as career decision-making self-efficacy and career choice anxiety.

Social support refers to the information, emotional comfort, material assistance, and self-trust obtained from personal relations (Revenson and Gibofsky, 1995). In addition, perceived social support is defined as a perception of how resources can function as a buffer against stressful events (Zimet et al., 1988) and consist of three dimensions: family, friends, and significant others. When people receive social support from family, friends, and significant others, they feel secure and companionable in their surroundings (Bozo et al., 2009). Thus, those who perceive their social relations as supportive are very likely to experience good results in life (Cohen and Wills, 1985). On the other hand, the perception of low social support not only lowers emotional well-being but also brings about mental health problems (Caserta et al., 2016).

Although research on the SCCT model has emphasized the impact of contextual barriers in the career development, Lent et al. (2000) called for consideration of supports as well as barriers in models exploring career development. In a SCCT framework, social support is defined as *contextual affordance* that helps career choice and development (Gushue and Whitson, 2006). Accordingly, we examined perceived social support as having the potential to influence career decision self-efficacy and career choice anxiety in this study.

In previous studies, Gushue and Whitson (2006) examined the influence of parent/teacher support on career decision self-efficacy and career outcome expectations in a sample of African–American high school students using the SCCT paradigm. Schultheiss et al. (2001) insisted that the social support that undergraduates receive from their family members was related to their career development. In addition, Patel et al. (2008) claimed that the family and peer support perceived by teenagers is a meaningful predictor of their career decision-making self-efficacy. Cohen and Wills (1985) argued that those who perceive their social relations to be supportive experience good results in life. Schultheiss et al. (2001) demonstrated that social support from family members was strongly related to career development and that it could be applied as an accelerating or buffering factor in career development. In qualitative studies (Kracke, 2002; Dietrich and Kracke, 2009), parental support and a children-centered parenting style were found to have a positive relationship with the career exploration of teenagers. Social support had similar effects on adolescents (Hirschi et al., 2011), high school students (Creed et al., 2009), and jobseekers (Zikic and Klehe, 2006).

Theoretically, social support may impact self-efficacy and judgment. In other words, individuals with high social support tend to be more confident in their lives and exhibit good judgment regarding what they should do. Gushue and Whitson (2006) empirically proved that both parental and teacher support were positively associated with career decision confidence in a population of high school students. Further, positive feedback from the parents and teachers boosted their self-confidence for career decision-making. Taken together, these seem to indicate that individuals with high social support are more confident in career decision-making compared with those with low social support. In this study, therefore, we expected that the interaction between positive affect and social support would impact career decision-making confidence. Given that social support helps career decision-making and development, high social support would strengthen the influence of positive affect on career decision-making self-efficacy. Therefore, we hypothesized as follows:

Hypothesis 3: Social support will moderate the relationship between positive affect and career decision-making self-efficacy such that the relationship would be stronger in individuals with high social support than in those with low social support.

Social support has been considered as a moderating variable in a variety of studies, and the buffer effect of social support has been extensively identified in various ages and circumstances. For example, social support moderated university students’ stress and assignment performance (Rees and Freeman, 2009). It had a moderating effect on adverse psychological outcomes such as stress, depression, and anxious emotions in a study that targeted German steel industry workers (Frese, 1999) and buffered the negative effect of activities of Daily Living performance on depression in the elderly (Hashimoto et al., 1999).

In the present study, we were also interested in the effect of social support as a moderating variable. In Hypothesis 2, relative to individuals with low positive affect, we expected those with high positive affect to experience less anxiety regarding their career decisions by inducing memories and beliefs related to success (Bandura, 1977). With social support from significant people, the effect of positive affect on anxiety reduction would be augmented. This is because social support may have a buffering effect on anxiety for individuals who are under stress about making a career decision. Given that social support helps the regulation of anxiety, it would augment the influence of positive affect on career choice anxiety. In other words, high social support and positive affect may induce memories related to success, leading to the recognition of self-worth and sense of stability, which, in turn, would lead to less career choice anxiety. Thus, we hypothesized as follows:

Hypothesis 4: Social support will moderate the relationship between positive affect and career choice anxiety such that the relationship would be stronger in individuals with high social support than in those with low social support.

### Influence of Future Time Perspective on Career Decision-Making Self-Efficacy and Career Choice Anxiety

Recently, future time perspective has been known as one of the important factors influencing individuals’ career decision (Husman et al., 2016). Future time perspective refers to a cognitive-motivational construct that represents one’s sense of purpose for the future (Carstensen, 2006; Zacher and Frese, 2009; Park and Jung, 2015). Future time perspective has been empirically investigated related to career decision processes such as career decision-making self-efficacy (Walker and Tracey, 2012; Jung et al., 2015) and career choice anxiety (Jung et al., 2015). However, no study has been conducted to examine its impact on daily career decision-making self-efficacy and daily career choice anxiety. The current study aimed to investigate those relationships within the SCCT framework.

The present study expected that future time perspective would be related to career decision-making self-efficacy. When people have high future time perspective, they tend to perceive their future to be salient. This leads people to set goals and generate strategies for attaining those goals (Strauss et al., 2012). Furthermore, individuals with future time perspective showed active establishment of their goals and expectations, efficient regulation of their behaviors, and persistent evaluation of their performance (Husman and Shell, 2008). As a corollary, people would feel strong confidence in their career decision-making. Prior studies empirically examined whether future time perspective was related to career decision-making self-efficacy (Walker and Tracey, 2012; Jung et al., 2015). The current study examined the impact of future time perspective on daily degree of career decision-making self-efficacy.

We believed that future time perspective would reduce career choice anxiety by promoting individuals’ confidence about their career choices. More specifically, individuals with high future time perspective can increase confidence and undermine the anxiety for their career choice by assisting individuals to focus on their goals in the future (Jung et al., 2015). Prior studies uncovered that future time perspective was negatively associated with career choice anxiety (Walker and Tracey, 2012; Taber, 2013; Jung et al., 2015). Aligned with the previous studies, we expected that future time perspective would reduce career choice anxiety measured daily. Thus, we expect as follows:

Hypothesis 5: Future time perspective will be positively associated with daily career decision-making self-efficacy.
Hypothesis 6: Future time perspective will be negatively associated with daily career choice anxiety.

### Influence of Self-Esteem on Career Decision-Making Self-Efficacy and Career Choice Anxiety

Self-esteem, the evaluative component of self-knowledge, is defined as how much people think of themselves as worthy (Baumeister et al., 2003). By definition, it is related to the degree of liking or disliking oneself (Lent and Fouad, 2011). Self-esteem and self-efficacy are highly correlated, but they are two distinct concepts (Chen et al., 2001; Lent and Fouad, 2011). Regardless of the belief about one’s capabilities, skills, and other characteristics, those with high self-esteem feel good about themselves whereas those with low self-esteem feel bad about themselves even when perceiving themselves to be competent (Chen et al., 2004). Regardless of the belief about one’s capabilities, skills, and other characteristics, those with high self-esteem feel good about themselves whereas those with low self-esteem feel bad about themselves even when perceiving themselves to be competent (Chen et al., 2004). Self-esteem is an important variable that affects various aspects of life, including occupation and job performance. Core self-assessments including self-esteem were meaningful variables that explained career decision-making self-efficacy (Betz et al., 1996). In other words, those with a high perception of self-esteem had a high tendency to aim for actions and proficiency, resulting in improved career decision-making self-efficacy. In the current study, we investigated the relationship between global self-esteem and daily career decision-making self-efficacy. Based on the previous studies, the present study anticipated that people with high self-esteem would have higher daily career decision-making self-efficacy than those with low self-esteem.

Self-esteem is generally related to emotional reactions, especially anxiety or avoidance affective processes (Baumeister et al., 2003). According to the dimensional theory of affect, individuals with high self-esteem may feel less anxiety (Brown and Marshall, 2001). Mineka et al. (1998) found that self-esteem was related to anxiety, a negative affect state. This indicates that people with low self-esteem are likely to experience greater anxiety with regard to career decision-making in daily life. Considering these, the following hypotheses were proposed in the present study:

Hypothesis 7: Self-esteem will be positively associated with career decision-making self-efficacy.
Hypothesis 8: Self-esteem will be negatively associated with career choice anxiety.

**FIGURE 1:**
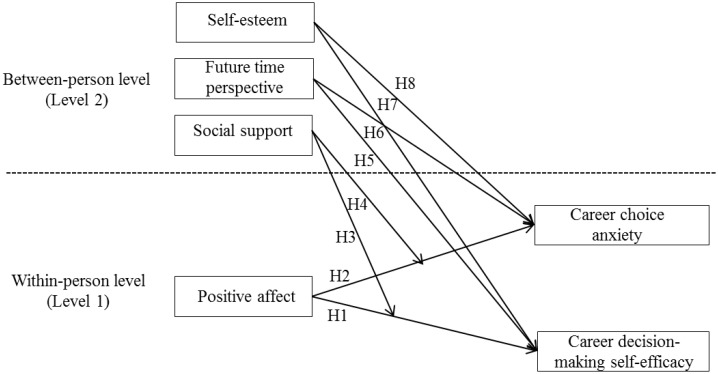
The overarching model and hypotheses.

All the hypothesized relationships examined in the present study have been illustrated in **Figure 1**.

## Materials and Methods

### Participants and Procedure

The sample size for this study was determined based on Kuppens et al.’s (2012) study in which 79 participants rated their feelings for 14 consecutive days. In the present study, 128 university students who took the “Introduction to Psychology” course at a university in Korea responded to the baseline survey. Of the students who responded to the first questionnaire, 23 refused to participate in the 21-day daily questionnaire. Therefore, the final sample comprised 105 participants. Of these, 40 participants were male (38.1%), and 65 were female (61.9%). Ages ranged from 18 to 33 years (*M* = 21.05 years, *SD* = 2.82 years). The participants included 35 first-year students (33.3%), 12 sophomores (11.4%), 44 juniors (41.9%), and 12 seniors (13.3%). Students were able to receive course credits by participating in the baseline survey and the 21-day daily survey.

One of the researchers of this study explained the purpose and research procedures to the participants in an introductory session. The participants were instructed to report their daily affect, career decision-making self-efficacy, and career choice anxiety through their smartphones or personal computers for 21 consecutive days. The students who agreed to do it took part in the study. The participants were asked to record their daily ratings using a website link that was sent in a text message at 9 o’clock every night. Those who did not register their responses were reminded to do so through an additional text message at 10 PM. Ratings were recorded on the same day, and, if participants missed 1 day, they were asked to rate the same thing the next day. After the introductory session, an initial survey was conducted. In the baseline survey, the participants were asked to respond about the following variables: self-esteem, social support, gender, and age. Their cell phone numbers were collected to send them text messages for the daily survey. The survey began in mid-March and finished in mid-April.

Daily rating began 1 week after the introductory session, and the contents of the text messages were the same as those used in the study by Park (2015). On the first day of the daily rating, the following message was sent: “Today is the first day of reporting your affective experiences and career decision-making process. The daily survey will take place for 21 consecutive days, and you will receive a text message at 9 PM. Log in to the website linked to the text message, and rate the items according to the instructions. Rate your affect, career decision-making self-efficacy, and career choice anxiety at the moment.” The total number of participants was 105, and 1,495 daily ratings were analyzed.

This study was conducted in accordance with the Declaration of Helsinki and was approved by Korea University’s review board. All participants gave written informed consent in accordance with the Declaration of Helsinki.

### Measures

#### Positive Affect

The experiences of positive affect throughout a day were measured using nine items from the four quadrants of the core affect scale (Kuppens et al., 2007). These items were translated into Korean and had been used in the study by Park (2015). The nine affective adjectives are happy, alert, proud, enthusiastic, excited, calm, satisfied, peaceful, content, and relaxed. The degree to which each affect was experienced in a day was rated on a seven-point Likert-type scale (1 = Not at all to 7 = Very much). The score for each day was computed by adding the ratings for all nine affects, and higher scores meant that more positive affect was experienced throughout the day. The average Cronbach’s alpha value over the 21-day study period was 0.92.

#### Career Decision-Making Self-Efficacy

We used a 25-item questionnaire that is a validated short-form of the career decision self-efficacy scale developed by Betz et al. (1996). The career decision self-efficacy scale comprises five items on four subordinate factors: accurate self-appraisal, gathering occupational information, goal selection, and making plans. The present study used five items—one item for each subordinate field—for daily measurement over the 21-day period. The following items were used: “Choose a major (field of study) or career that fits your interests,” “Persistently work on your major or career goal even when you get frustrated,” “Determine the kind of lifestyle you like,” “Identify the employers relevant to your career possibilities,” and “Determine the steps to take if you are having academic troubles with your major.” Each item was measured on a seven-point Likert-type scale (1 = no confidence at all to 7 = complete confidence), and a high score indicated high self-efficacy toward career decision-making. Lee (2001) used this scale for her study after translating into Korean. In the study by Betz et al. (1996), the Cronbach’s alpha of the CDSE-short form was 0.94, and the average Cronbach’s alpha for the present 21-day study period was 0.88.

#### Career Choice Anxiety

To measure the degree of anxiety related to the process of career decision-making, six items from the revised version of Spielberger’s State-Trait Anxiety Inventory (STAI) were used (Spielberger et al., 1983). Marteau and Bekker (1992) developed and validated a six-item short-form of the STAI. The responses to three out of the six items were positively worded, such as “I feel calm” or “I am relaxed,” and the rest were negatively worded, such as “I am tense” or “I feel worried.” Prior studies have utilized similarly modified career scales (Fuqua and Hartman, 1983; Weinstein et al., 2002). The participants read the following statement in the daily survey: “Assess how you experience at this moment when you think about your career that is being determined or undetermined by you” and then responded to the six items mentioned above for 21 days. Respective items were measured using a seven-point Likert-type scale (1 = Totally disagree to 7 = Totally agree), and a high score indicated high anxiety in a situation related to career decision-making. The Cronbach’s alpha of the six items in the study by Marteau and Bekker (1992) was 0.95, and the average Cronbach’s alpha for the present 21-day study period was 0.78.

#### Future Time Perspective

We measured future time perspective using a 10-item scale designed by Carstensen and Lang (1996), which was validated in previous studies (Cate and John, 2007; Zacher and Frese, 2009). Participants rated the extent to which they agreed with each of the 10 items on a scale ranging from 1 (not at all) to 7 (to a very great extent). Sample items include the followings: “Many opportunities await me in my future,” “I expect that I will set many new goals in my future,” and “As I get older, I begin to experience time in my future as limited” (reverse coded). The Korean version was translated by Kim (2008), and Cronbach’s alpha for the scale was 0.80 in our study.

#### Self-Esteem

To measure self-esteem, the scale developed by Rosenberg (1965) and translated and validated by Lee and Won (1995) was used. This scale, which is one of the most widely used tools worldwide, comprises 10 items related to positive and negative assessments of oneself—e.g., “I am able to do things as well as most other people can” and “I certainly feel useless at times.” Respective items were measured using a five-point Likert-type scale (1 = Not at all to 5 = Very much), and a high score indicated high self-esteem. In the study by Schmitt and Allik (2005), which involved samples from 53 countries, the Cronbach’s alpha of the Korean sample was 0.83, and that in the present study was 0.86.

#### Social Support

To assess social support, the Multidimensional Scale of Perceived Social Support (MSPSS) developed by Zimet et al. (1988); the Korean version was translated and validated by Shin and Lee (1999). The MSPSS comprises 12 items measuring the social support perceived from three sources: family, friends, and significant others. “My family tries to help me,” “I have friends with whom I share joys and sorrows,” and “There is a special person around when I am in need” are examples of some items. Respective items were measured using a five-point Likert-type scale (1 = Not at all to 5 = Very much), and a high score indicated higher perceived social support from family, friends, and significant others. The Cronbach’s alpha of the scale in the study by Shin and Lee (1999) was 0.89, and that in the present study was 0.90.

### Data Analysis

This study collected data using a multilevel structure, including variables at both the within-person (Level 1) and between-person levels (Level 2). Therefore, hierarchical linear modeling (HLM; Raudenbush et al., 2004) was employed to analyze the multilevel data. In this study, the between-person variables (i.e., self-esteem, future time perspective, and social support) were centered at the grand mean while the within-person variables (i.e., positive affect, career decision-making self-efficacy, and career choice anxiety) were centered at each person’s mean, which excluded all between-person variance in the variables at the within-person level (Hofmann et al., 2000; Gavin and Hofmann, 2002). Future time perspective at the between-person level as a control variable was included because previous studies showed that it is related to career decision-making self-efficacy and career choice anxiety (e.g., Walker and Tracey, 2012; Jung et al., 2015). This study handled missing data with listwise deletion.

## Results

### Descriptive Statistics

**Table 1** presents the means, standard deviations, and correlations of the study variables. Before using listwise deletion for the missing data, we conducted Little’s missing completely at random test. The results showed that the missing values for intra-individual variables were not missing completely at random in our data [χ^2^(220) = 274.58, *p* < 0.01].

**Table 1:** The means, standard deviations, and the between-person and within-person correlations among study variables.

Variables	*M*	*SD*	1	2	3	4	5	6
Within variables								
(1) Positive affect	4.52	1.11	-					
(2) CDSE	4.93	1.05	0.49^∗∗^	–				
(3) CCA	3.92	0.97	-0.42^∗∗^	-0.51^∗∗^	–			
Between variables								
(4) Self-esteem	3.68	0.62	0.34^∗∗^	0.42^∗∗^	-0.22^∗∗^	–		
(5) Social support	3.36	0.52	0.28^∗∗^	0.36^∗∗^	-0.20^∗∗^	0.34^∗∗^	-	
(6) FTP	5.04	0.72	0.33^∗∗^	0.36^∗∗^	-0.33^∗∗^	0.46^∗∗^	0.52^∗∗^	–


*^∗∗^*p* < 0.01; CDSE, Career Decision-Making Self-Efficacy; CCA, Career Choice Anxiety; FTP, Future Time Perspective.*

The intraclass correlation coefficient was computed to determine if there was significant between-person variance to confirm if HLM is an appropriate method to analyze the data (Hofmann et al., 2000). To examine the stability of CDSE and career choice anxiety across 21 days, we explored the intra-variability of CDSE and career choice anxiety. The results showed that CDSE (γ = 5.00, *p* < 0.001) and career choice anxiety (γ = 3.85, *p* < 0.001) are varied as time lapse during 21 days. Regarding career decision-making self-efficacy, 78 percent of the variance resided between people (τ_00_ = 0.86, *p* < 0.001, σ^2^ = 0.24) while, for career choice anxiety, 67 percent of the variance resided between people (τ_00_ = 0.62, *p* < 0.001, σ^2^ = 0.29). The HLM analysis was deemed appropriate because the results showed that the between-person variance for both outcome variables was significant.

### Positive Affect, Career Decision-Making Self-Efficacy, and Career Choice Anxiety

To examine hypotheses 1 and 2, we regressed positive affect on career decision-making self-efficacy and career choice anxiety. The values of the Level 1 variables were centered with the individuals’ means to exclude between-individual variance. Accordingly, the estimates ensured strict within-person association (Ilies et al., 2006). As shown in **Table 2**, positive affect (at Time t) was positively associated with career decision-making self-efficacy (γ = 0.17, *p* < 0.001) and career choice anxiety (γ = -0.19, *p* < 0.001). Thus, Hypotheses 1 and 2 were supported.

**Table 2:** Hierarchical linear modeling results of the effect of positive affect on decision-making self-efficacy and career choice anxiety.

	CDSE		CCA	
		
	Model 1-1	Model 1-2	Model 2-1	Model 2-1
Intercept	5.01^∗∗∗^	4.99^∗∗∗^	3.83^∗∗∗^	3.85^∗∗∗^
Time index	0.00	0.00	0.00	0.00
Positive affect (Time t)		0.17^∗∗∗^		-0.19^∗∗∗^
Lagged positive affect (Time t-1)		0.00		-0.04^∗^


*The values of the Level 1 variables were centered with the individuals’ means to exclude between-individual variance.*

Additionally, we examine the lagged effects of positive affect on career decision-making self-efficacy and career choice anxiety to probe if it slightly delayed the effects on these variables. The lagged positive affect was centered with the mean of each individual to exclude between-person variance. The results show that lagged positive affect (Time t-1) was not related to career decision-making self-efficacy (Time t) (γ = 0.00, n.s.) whereas it was found to be negatively related to career choice anxiety (Time t) (γ = -0.04, *p* < 0.05). These results suggest that positive affect may not have a lasting effect on career decision-making self-efficacy but may do so on career choice anxiety.

### The Cross-Level Interaction Effects of Social Support on the Relationship of Positive Affect With Career Decision-Making Self-Efficacy and Career Choice Anxiety

We predicted that social support would moderate the relationship between positive affect and career decision-making self-efficacy such that the relationship would be stronger in individuals with high social support than in those with low social support (Hypothesis 3). As shown in **Table 3**, the results show that there was no significant interaction effect (γ = 0.02, n.s.). Therefore, the results failed to support Hypothesis 3.

**Table 3:** Hierarchical linear modeling results of the hypothesized effects on decision-making self-efficacy and career choice anxiety.

	CDSE		CCA	
		
Predictors	γ	*SE*	γ	*SE*
Intercept	5.00^∗∗∗^	0.08	3.84^∗∗∗^	0.07
Level 1 variables				
Time index	0.00	0.00	0.00	0.00
Positive affect	0.18^∗∗∗^	0.02	-0.21^∗∗∗^	0.02
Level 2 variables				
Future time perspective	0.13	0.12	-0.24^∗^	0.12
Self-efficacy	0.72^∗∗^	0.12	-0.55^∗^	0.24
Self-esteem	0.33^∗^	0.16	-0.11	0.15
Social support	0.34^∗^	0.16	-0.06	0.16
Interaction term				
Positive affect × Social support	0.02	0.03	-0.11^∗∗∗^	0.03


**FIGURE 2:**
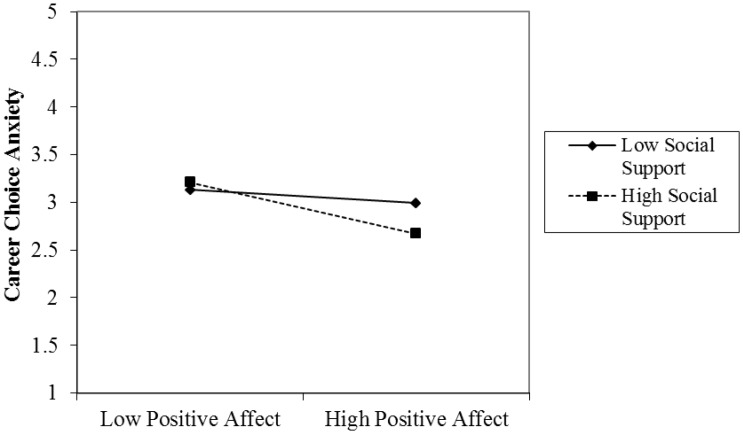
The moderating effect of social support on the relationship between positive affect and career choice anxiety.

Hypothesis 4 predicted that social support would moderate the relationship between positive affect and career choice anxiety such that the relationship would be stronger in individuals with high social support than in those with low social support. The interaction term of positive affect and social support on career choice anxiety was significant (γ = -0.11, *p* < 0.001). We then examined if this significant effect was consistent with the hypothesized trend using a simple slope test for cross-level interaction (Preacher et al., 2006). **Figure 2** graphically represents the interactive effect of these relationships. As predicted, positive affect had a stronger relationship with career choice anxiety when social support was high (+1 *SD*) than when it was low (-1 *SD*). The results show that the simple slope at +1 *SD* of social support was significant (*B* = -0.27, *SE* = 0.09, *t* = -2.92, *p* < 0.01) whereas the simple slope at -1 *SD* of social support was not significant (*B* = -0.11, *SE* = 0.06, *t* = -1.80, *p* > 0.05). Thus, Hypothesis 4 was supported.

### Self-Esteem, Future Time Perspective, Career Decision-Making Self-Efficacy, and Career Choice Anxiety

We predicted that self-efficacy would have a significant effect on both career decision-making self-efficacy and career choice anxiety (Hypotheses 5 and 6). Future time perspective was centered with the grand-mean. The results showed that future time perspective was not related to career decision-making self-efficacy (γ = 0.13, *p* > 0.05) and negatively to career choice anxiety (γ = -0.24, *p* < 0.05). Therefore, Hypotheses 5 was not supported but 6 were supported.

In Hypotheses 7 and 8, we proposed that self-esteem would have a significant effect on both career decision-making self-efficacy and career choice anxiety. The results showed that self-esteem was positively related to career decision-making self-efficacy (γ = 0.33, *p* < 0.05) whereas no relationship was found with career choice anxiety (γ = -0.11, *p* < n.s.). Therefore, Hypothesis 7 was supported, and Hypothesis 8 was not.

## Discussion

Based on SCCT, this study examined the influence of intra-individual variability (i.e., positive affect) and inter-individual differences in self-concept (i.e., self-esteem) on the variability in career decision-making self-efficacy and career choice anxiety. In addition, this study investigated the moderating effects of contextual influence (i.e., social support) on the relationship between the experience of positive affect and career decision processes (i.e., career decision-making self-efficacy and career choice anxiety) using a daily diary method for 21 consecutive days.

The following are the main results of this study. First, positive affect was associated positively with career decision-making self-efficacy and negatively with career choice anxiety. This means that higher positive affect experienced daily corresponded with higher career decision self-efficacy; lower positive affect experienced corresponded with higher career choice anxiety. Several previous studies have identified that the experience of positive affect at the inter-individual difference is associated with career-related self-efficacy (Hammond et al., 2010), job confidence (Larson and Borgen, 2006), clarity in job exploration, and job exploration self-efficacy (Côté et al., 2006). The current study is the first to investigate the relationship between positive affect and career decision-making self-efficacy and career choice anxiety at the intra-individual level. These results are aligned with previous studies finding that positive affect anticipates the changes within individuals at the career engagement level and that highly activated positive feelings promote self-directed objective control related to career decision-making (Hirschi and Freund, 2014). It seems that individuals are ready to set goals and perform a variety of actions for career management when positive affect improves career decision-making self-efficacy, lowers career choice anxiety, and promotes engagement in activities that enhance career resources.

Second, social support (at time 1) moderated the relationship between positive affect and career choice anxiety in that the relationship was stronger when individuals had high rather than low social support. More specifically, social support had a synergy effect with positive affect in reducing career choice anxiety. Higher perception of social support indicates that the individual receives assistance for career development in several forms. Therefore, a high level of social support will strengthen the influence of positive affect on reducing career choice anxiety. However, there was no significant interaction effect of social support on the relationship between positive affect and career decision-making self-efficacy.

Third, self-esteem was related positively to career decision-making self-efficacy and negatively to career choice anxiety. That is, individuals who have high self-esteem tend to be confident about their career decision-making processes and have less anxiety regarding their career choices.

### Theoretical and Practical Implications

The current study has some theoretical and methodological implications. First, we identified the impact of positive affect experienced in daily life on the daily degrees of career decision-making self-efficacy and career choice anxiety. Most previous studies on the role of affect in career decision-making tended to regard affect as a general and stable characteristic (Judge and Larsen, 2001). Therefore, they investigated the inter-individual differences and measured emotional state just once with a cross-sectional design. However, emotions vary often and are not stable (Kidd, 2004; Hartung, 2011). This indicates that, to understand the accurate and microscopic influence of affect on career decision-making process, we must evaluate and measure individuals’ experiences of affect repeatedly over a specific period (Hartung, 2011). We investigated the influence of positive affect experienced in daily life on the daily degrees of career decision-making (i.e., self-efficacy and career choice anxiety) by measuring these variables repeatedly over 3 weeks. As a result, we could reflect the variability in affective events experienced (i.e., intra-individual differences). The daily reconstruction method used in our study has more ecological validity than a one-shot survey or cross-sectional study (Reis and Judd, 2000).

Second, our study supports SCCT, in which affectivity is regarded as one of the individual characteristics that influence career-related self-efficacy and outcome expectations (Thompson et al., 2017). In addition, this study broadens SCCT in that positive affect in daily life influences changes in career decision-making self-efficacy and career choice anxiety. Positive affect had significant influence on positive (i.e., self-efficacy) and negative dependent variables (i.e., anxiety).

Third, social support (at time 1) had a synergy effect with positive affect to reduce career decision anxiety. This indicates that social support does not need to occur every day to manage career related anxiety whereas positive affect should occur daily.

Finally, self-esteem (at time 1) as a general and stable characteristic had significant effect on daily basis measures such as career decision-making self-efficacy and career choice anxiety. This result confirms SCCT in that self-esteem and career decision-making self-efficacy are distinctive constructs. Betz et al. (1996) found small correlation between these two concepts, but our result shows meaningful relationships. Furthermore, we broadened SCCT by figuring out the accurate relationship between global self-esteem and daily measured career decision-making self-efficacy. In addition, our study expanded the dimensional theory of affect (Mineka et al., 1998; Brown and Marshall, 2001) by investigating the influence of self-esteem on anxiety daily based on a daily fluctuation.

Our study has some practical implications. First, this study showed that self-esteem is an important variable in the career decision-making process. This core self-assessment is a flexible variable that can be improved through training, coaching, and counseling. The present findings suggest that it is necessary to focus on the improvement of self-esteem when intervening for career decision-making development (Di Fabio et al., 2012). Second, this study has an implication in the field of career counseling psychology, as utilizing a diary method. Most of scholars in counseling psychology traditionally focused on between subject designs. This study might extend methodology to explore issues in the field of counseling psychology. A diary study can uncover clients’ psychological state based on daily fluctuation that would help more ecologically understand the client. Finally, the result that daily positive affect influenced daily career decision-making self-efficacy and career choice anxiety provides insights for career counseling. According to Bandura (1997), counselors must consider positive affect rather than improving ability or information in the interventions for career development. That is, clients can make a favorable assessment of their own capabilities with the experiences of positive affect. As a result, they can be more confident and less anxious about their career decision-making. Therefore, career development counselors must improve the positive affect of clients by helping them to develop trust in themselves to establish challenging but obtainable goals (Emmons, 1986).

### Limitations and Future Direction

Despite the contributions of this study and the utility of its findings, it has some limitations. First, this study was not able to include control variables related to the career decision-making process. For instance, academic scores or career development support system within an organization are important variables affecting the career decision-making of university students, but this study could not control their effects. Therefore, since the results of this study could have been confounded by these variables, a study including more extensive control variables can be conducted in the future.

Second, this study analyzed the effects of the experiences of positive affect on career decision-making self-efficacy and career choice anxiety, but they cannot be interpreted as cause-and-effect relationships. Therefore, future studies should be designed to identify the cause-and-effect relationships among these variables. For example, if a study on a counseling intervention that enhances the experience of positive affect results in the improvement of career decision-making self-efficacy and the decline of career choice anxiety, it can be clear evidence explaining the cause-and-effect relationship between the two.

Third, it is necessary to conduct studies on the role of the career decision-making process by classifying social support according to dimensions. For instance, social support can be classified as perceived and received social support (Schwarzer et al., 2003). This study did not consider the multi-dimensional characteristics of social support and merely examined the perception of social support experiences. However, it would be more meaningful for future studies to identify whether future expectations of or received support are salient in predicting higher positive affect.

Fourth, the results of this study that career decision-making self-efficacy and career choice anxiety can change over 21 days is very meaningful. However, the studies investigating fluctuation of career decision-making variables are at an early stage, and subsequent studies should be conducted to confirm the results. In future studies, a cross-lagged model could be used to identify daily variation and longitudinal influence between variables.

Finally, it is difficult to say that the current study tested the entire career decision process. Rather, the current study focused on career decision-making self-efficacy and career choice anxiety predicting career decision difficulty or career indecision. However, to have a practical implication for those who have career decision difficulty, it is necessary to study career indecision and career decision difficulty in future studies.

## Author Contributions

All authors listed have made a substantial, direct and intellectual contribution to the work, and approved it for publication.

## Conflict of Interest Statement

The authors declare that the research was conducted in the absence of any commercial or financial relationships that could be construed as a potential conflict of interest.
